# Impact of Urban Slum Residence on Coverage of Maternal, Neonatal, and Child Health Service Indicators in the Greater Accra Region of Ghana: an Ecological Time-Series Analysis, 2018–2021

**DOI:** 10.1007/s11524-023-00812-0

**Published:** 2023-11-16

**Authors:** Duah Dwomoh, Samuel Iddi, Seth Kwaku Afagbedzi, Natalia Tejedor-Garavito, Winfred Dotse-Gborgbortsi, Jim Wright, Andrew J Tatem, Kristine Nilsen

**Affiliations:** 1 Department of Biostatistics, School of Public Health, University of Ghana, Accra, Ghana; 2 Department of Statistics, School of Physical and Mathematical Sciences, University of Ghana, Accra, Ghana; 3 Chronic Disease Management Unit, African Population and Health Research Center (APHRC), Nairobi, Kenya; 4 School of Geography and Environmental Science, University of Southampton, Southampton, UK; 5 WorldPop, School of Geography and Environmental Science, University of Southampton, Southampton, UK; 6 Department of Social Statistics and Demography, University of Southampton, Southampton, UK

**Keywords:** Urban slum, Ecological time series, Maternal neonatal and child health outcomes

## Abstract

Among other focus areas, the global Sustainable Development Goals (SDGs) 3 and 11 seek to advance progress toward universal coverage of maternal, neonatal, and child health (MNCH) services and access to safe and affordable housing and basic services by 2030. Governments and development agencies have historically neglected the health and well-being associated with living in urban slums across major capital cities in sub-Saharan Africa since health policies and programs have tended to focus on people living in rural communities. This study assessed the trends and compared inequities in MNCH service utilization between slum and non-slum districts in the Greater Accra region of Ghana. It analyzed information from 29 districts using monthly time-series Health Management Information System (HMIS) data on MNCH service utilization between January 2018 and December 2021. Multivariable quantile regression models with robust standard errors were used to quantify the impact of urban slum residence on MNCH service utilization. We assessed the inequality of MNCH coverage indicators between slum and non-slum districts using the Gini index with bootstrapped standard errors and the generalized Lorenz curve. The results indicate that rates of vaccination coverage and antenatal care (ANC) attendance have declined significantly in slum districts compared to those in non-slum districts. However, skilled birth delivery and postnatal care (PNC) were found to be higher in urban slum areas compared to those in non-urban slum areas. To help achieve the SDGs’ targets, it is important for the government of Ghana and other relevant stakeholders to prioritize the implementation of effective policies, programs, and interventions that will improve access to and utilization of ANC and immunization services among urban slum dwellers.

## Introduction

The United Nations 2030 Agenda for Sustainable Development includes 17 Sustainable Development Goals (SDGs). One of these goals, SDG 11 (“Make cities and human settlements inclusive, safe, resilient and sustainable”), has an explicit urban focus [1]. Urbanization is an ongoing major global trend. According to the World Health Organization (WHO), more than 55% of the world’s total population resides in urban areas, and this proportion is projected to increase to 68% by 2050 [2]. In 2018, three times as many urban dwellers were estimated to live in less developed regions compared to more developed regions (3.2 billion versus 1 billion) [1]. These recent estimates and projections make it increasingly urgent to understand and address challenges in rapid urban population growth, particularly in low- and middle-income countries (LMICs).

According to WHO, most people living in urban areas still experience inadequate housing, transport, sanitation, and waste management, poor air quality, lack of quality health care, limited access to social welfare services, and few economic opportunities [2]. A recent study in Nepal assessed the utilization of maternal health services among women residing in slum areas and concluded that antenatal care (ANC) service utilization was satisfactory whereas postnatal care (PNC) and family planning service utilizations were very poor [3]. Another study from that country reported similar findings and emphasized the need to implement interventions geared toward improving the utilization of PNC services in urban slums [4].

While urbanization can enhance economic growth, poverty alleviation, and development, potential gains are largely dependent on its pattern, service delivery quality, and infrastructure development that are required to keep pace with urban population increase [5]. In many cases, the urban poor may not only lack the financial wherewithal to afford decent living conditions, but may also experience limited access to some essential health services, including maternal, neonatal, and child health (MNCH) services, as well as opportunities for employment and social development [6]. WHO has placed high priority on enhancing the utilization of MNCH services, including because inferior access to these services among people living in urban slums poses a threat to progress made and may hinder the achievement of critical SDG targets, including reducing maternal mortality to below 70 per 100,000 live births, decreasing neonatal mortality to at least 12 per 1000 live births, and lowering under-5 mortality to at least 25 per 1000 live births. Globally, pregnant women who live in urban slum communities are at a high risk of maternal deaths due in part to limited access to health facilities [7], and children tend to have much poorer health outcomes, including in regard to childhood illnesses, malnutrition, and mortality than children in other residential domains, including those living in rural areas [8]. However, this urban slum disadvantage is often hidden. Statistics show that overall, people residing in urban areas tend to be better off in terms of their general well-being and have a greater choice of health services than their rural counterparts [9]. However, these statistics typically obscure disparities in most urban areas because the urban poor and their unmet needs are not visible within per capita averages. Population-based surveys often mask disparities in urban areas because sample sizes are too small to show disaggregation by wealth status or geographic location. The health and general well-being of poor people living in slum areas in large cities in Africa have deteriorated as most government policies and other community-based interventions often overlook this population and focus on people living in rural communities [10]. Throughout most urban slums in Africa, there are also a lack of health insurance, inadequate and poor accommodation, and water and sanitation challenges [11]. And as people migrate into slums, continuity of care in vital MNCH areas such as ANC, facility-based deliveries, PNC, and vaccination coverage suffers.

There is a paucity of evidence regarding the impact of living in slums on health in sub-Saharan Africa [12]. To the best of our knowledge, no study has quantified the impact of urban slum–based intercity inequalities on service utilization in sub-Saharan Africa using routine Health Management Information System (HMIS) data that continuously capture utilization of health services and the health status of urban slum dwellers in large cities. Admittedly, a lot is known about intra-urban inequalities in Greater Accra, which means there is much contextual knowledge to aid the interpretation of findings from routine health systems data. To make informed decisions to improve the health of populations living in slum areas, it is essential to generate and track reliable estimates of MNCH outcomes and service utilization in both slum and non-slum areas and to further quantify the impact of living in urban slum districts on selected outcomes and coverage indicators. This study assessed the impact of urban slum residence on the coverage of four MNCH service indicators (ANC, skilled birth attendance, PNC, and vaccination) in the Greater Accra region of Ghana.

## Methods

### Study Area

This study is an ecological time-series study across 29 districts in the Greater Accra region of Ghana between 2018 and 2021. Greater Accra is the region where Ghana’s national capital, Accra, is located. The region has health facilities including teaching hospitals, regional hospitals, university hospitals, district hospitals, psychiatric hospitals, polyclinics, clinics, maternity homes, health centers, private health facilities, NGO-run facilities, Christian Health Association of Ghana facilities, and community health posts. In this study, the unit of observation is the population of children and women using health services across the 29 administrative districts. The Greater Accra region has a population of 5.5 million persons according to the 2021 population and housing census [13]. The population of Ghana has not only grown but has also experienced rapid urbanization in the past several decades, particularly in the Greater Accra and Ashanti regions. Today, more than half of the country’s population resides in urban areas [14]. According to a UN-Habitat report, the proportion of the urban population living in slum households in Ghana was 30.4% as of 2018. Using 2010 national census data and UN-Habitat’s definition of slums, the Accra Metropolitan Assembly identified 78 informal settlements and hotspots in Accra [15]. According to the World Bank, approximately 37.4% of people who live in Ghana’s urban regions live in slums [16].

### Data Sources

The unit of analysis in this study is the district where the facilities are located, because of the difficulty of identifying catchment populations (unstable denominators) for individual facilities at the sub-district level in the Greater Accra region. In addition, there were no reliable data on population size at the sub-district level. We used routine Health Management Information System (HMIS) data on MNCH health outcomes and service utilization as clients interact with health services at health facilities or during outreach services. Routine HMIS is a comprehensive solution for the reporting and analysis needs of district health administrations and health facilities at every level. Healthcare workers at facilities collect the data and input aggregated reports via the district HMIS database in Ghana. The system receives data from government, NGOs/mission facilities, and private health facilities and has been operational since 2012. We obtained monthly data on the indicators from the district HMIS between 2018 and 2021 from the Office of Policy, Planning, Monitoring, and Evaluation Division of the Ghana Health Service and merged the data with slum information at the district level. The data on service utilization of ANC, skilled birth attendance (SBA), and PNC used were monthly aggregates of women receiving maternal health services at different health facilities. The vaccination coverage indicators used were aggregates of services provided at health facilities and through outreach programs provided by community health nurses.

Slum areas were identified by triangulating field survey, spatial, and census data. The data on slum locations across Greater Accra were derived from the synthesis of data obtained from the field in 2021. Also used were Ghana Statistical Service slum-delineated data for 2021 and a literature review of similar slum identification in Greater Accra using census, survey, and remotely sensed and GPS-located data [17, 18]. The data on the population size and population projections for districts used the national population and housing census 2010 and were obtained from the Ghana Statistical Service.

### Health Service Coverage Indicators

The primary service coverage measures include ANC attendance, skilled birth attendance, PNC attendance, and vaccination coverage for Bacillus Calmette-Guérin (BCG), oral polio, measles, and pentavalent 1 (Penta1) vaccine at the district level (Table 1).

**Table 1:** Health service coverage indicator definitions

Indicator	Definition	Numerator	Denominator	Source of denominator
BCG vaccination coverage	The proportion of children under 1 year receiving BCG vaccine	Number of children under 1 year receiving the BCG vaccine in the period	Number of children under 1 year (estimated as 4% of the population)	Ghana Statistical Service from the 2010 Population and Housing Census adjusted for monthly population growth rate
Oral polio vaccination	The proportion of children under 1 year receiving oral polio (OPV1) vaccine	Number of children under 1 year receiving the OPV1 vaccine in the period	Number of children under 1 year (estimated as 4% of the population)	Ghana Statistical Service from the 2010 Population and Housing Census adjusted for monthly population growth rate
Pentavalent vaccination	The proportion of children under 1 year receiving Penta1 vaccine	Number of children under 1 year receiving the Penta1 vaccine in the period	Number of children under 1 year (estimated as 4% of the population)	Ghana Statistical Service from the 2010 Population and Housing Census adjusted for monthly population growth rate
Measles-rubella coverage	The proportion of children under 1 year receiving measles-rubella vaccine	Number of children under 1 year receiving the measles-rubella vaccine in the period	Number of children under 1 year (estimated as 4% of the population)	Ghana Statistical Service from the 2010 Population and Housing Census adjusted for monthly population growth rate
ANC coverage	The percentage of pregnant women receiving ANC during pregnancy (at least once)	Total number of ANC registrants in a specified period	Number of expected pregnancies of the catchment area within the specified period (estimated as 4% of the population)	Ghana Statistical Service from the 2010 Population and Housing Census adjusted for monthly population growth rate
Skilled birth attendance	The percentage of deliveries conducted by skilled attendants (midwives, nurses, and doctors).	The number of deliveries supervised by doctors or nurses in the specified period	Number of expected pregnancies (estimated as 4% of the population)	Ghana Statistical Service from the 2010 Population and Housing Census adjusted for monthly population growth rate
PNC coverage	The percentage of PNC registrants seen after delivery for mothers and newborn babies	Number of PNC registrants (within 48 h)	Number of expected pregnancies (estimated as 4% of the population)	Ghana Statistical Service from the 2010 Population and Housing Census adjusted for monthly population growth rate

Abbreviations: *ANC*, antenatal care; *PNC*, postnatal care; *BCG*, Bacille Calmette-Guérin vaccination

### Defining Slums in the Greater Accra Region

An essential component of the study was identifying the number of slums in a district. UN-Habitat 2004 defines a slum household as a group of individuals living under the same roof in an urban area who lack one or more of the following: (1) durable housing of a permanent nature that protects against extreme climate conditions; (2) sufficient living space, which means not more than three people sharing a single room; (3) easy access to safe water in sufficient amounts at an affordable price; (4) access to adequate sanitation in the form of a private or public toilet shared by a reasonable number of people; and (5) security of tenure that prevents forced evictions [1].

In this study, we based the determination of slums in Greater Accra on the UN-Habitat definition of a slum. The slum areas were identified by triangulating three data sources: (1) evidence from literature based on the UN-Habitat definition in the last two decades; (2) a listing of slums in Greater Accra from the Ghana Statistical Service (GSS); and (3) a field survey. From the literature, we extracted maps of Accra slums from two published manuscripts [17, 18]. These Accra slum maps were digitized, georeferenced, and compared to establish the location of slums in Accra. The list of slum locations obtained from the GSS was geocoded and mapped, which transformed place names or addresses into spatial data. These two data sources were overlaid to be sure that the borders matched and further validated the slum map based on this overlay through site visits. That is, the research team validated the existence of slums in the locations identified in the literature and the list of slum locations obtained from the GSS through field visits.

The final judgment of what could be defined as slum locations was decided by the team based on the UN-Habitat definition, and they took into consideration that not all slums are homogeneous and not all slum dwellers experience the same degree of deprivation. The degree of deprivation depends on how many of the five conditions that define slums, as per UN-Habitat, are prevalent within a slum household. The final list of slums identified includes households that experience at least two shelter deprivations.

**Fig. 1:**
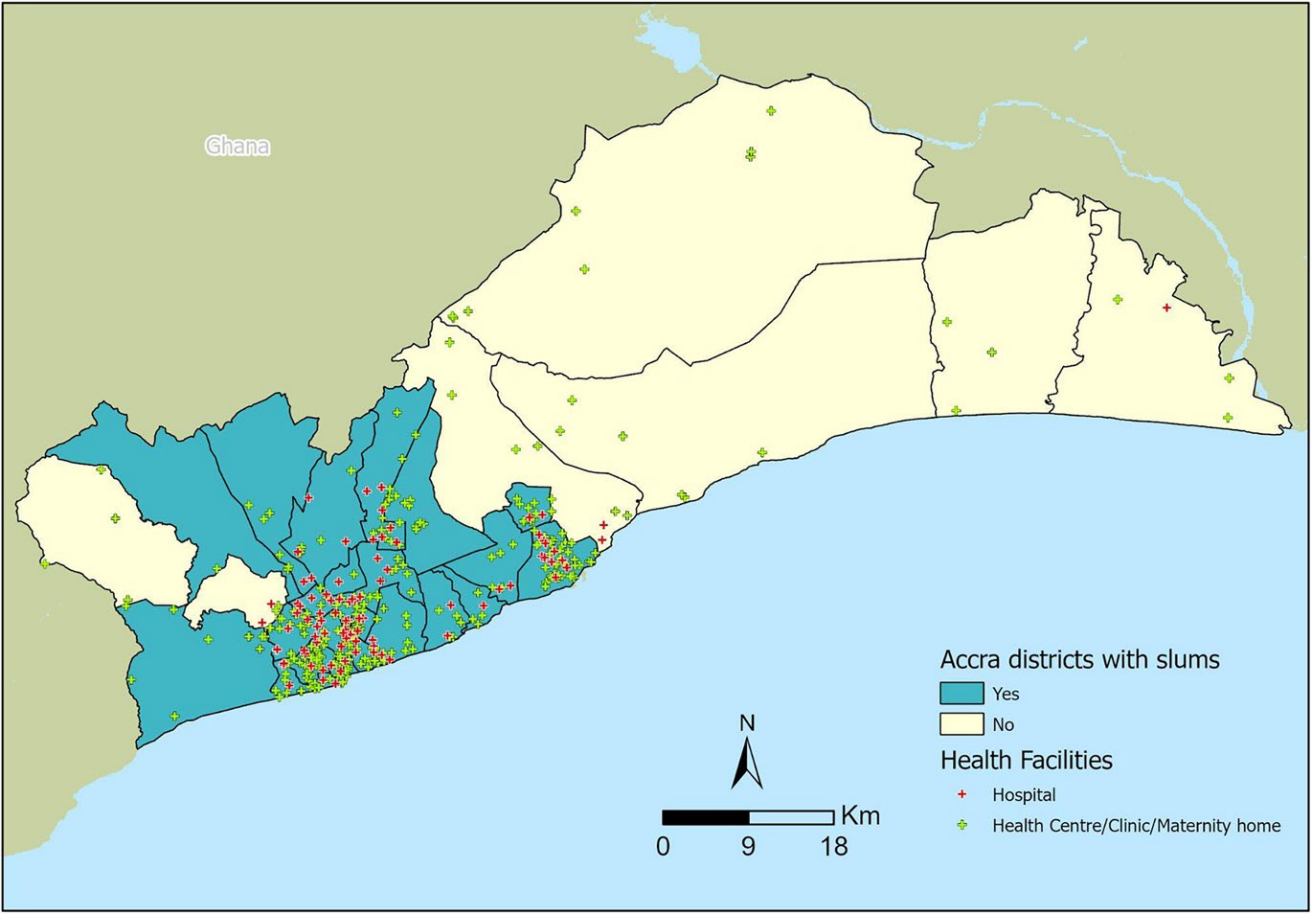
Districts within the Greater Accra region with slums/no slums and type of health facilities

Because districts were used as the unit of analysis in this study, the districts in Greater Accra were categorized into slum and non-slum districts. A district was designated as containing slums if it met one or both of the following conditions: (1) sufficient intersection with the slum areas derived from the literature and UN-Habitat definition (i.e., at least one-quarter or more of the households in the district), and (2) at least one town from the GSS list of towns. A total of 22 out of 29 districts in the Greater Accra region were classified as containing slums, and the remaining seven were considered non-slum districts (Fig. 1).

### Statistical Analysis

Descriptive summary measures such as median, 25th and 75th percentile, mean, standard deviation, and range were used to describe the service coverage measures of interest. In addition, time-series tools were used to explore the distribution of the coverage measures and identify the underlying coverage trends between the urban slum and urban non-slum districts, seasonal patterns, and outliers.

Initial data exploration showed the coverage measures were heavily skewed. Therefore, we quantified the impact of living in urban slum districts on MNCH outcomes and service utilization using the quantile regression (least-absolute-value models, median absolute deviation, and minimum *L*1-norm) models with a robust standard error. Furthermore, we adjusted for seasonal deviation and linear time trends. The quantile regression is a natural extension of the ordinary least square (OLS) regression model that is used when the conditions of OLS regression are not met (i.e., linearity, homoscedasticity, independence, or normality). The quantile regression model equation for the *τ*th quantile is given as follows:

$${Q}_{\tau}\left({y}_{ij}\right)={\beta}_0\left(\tau \right)+{\beta}_1\left(\tau \right)\textrm{slum}+{\beta}_2\left(\tau \right)\textrm{month}+{\beta}_3\left(\tau \right)\left(\textrm{year}\right)+{\beta}_4\left(\tau \right)\left(\textrm{facilities}\right)+{\beta}_5\left(\tau \right)\textrm{COVID}+{\beta}_6\left(\tau \right)\textrm{location}+{\beta}_7\textrm{OPD}+{\beta}_8\textrm{Pop}+{\varepsilon}_{ij}\left(\tau \right),i=1,\dots, n$$

where *y*_*ij*_ is the *i*th month observation for the *j*th districts. All the multivariable models adjusted for seasonality in month and year fixed effect, the impact of COVID-19 (a binary indicator indicating observations before and after the onset of COVID-19), total outpatient department (OPD) attendance, number of health facilities in the district (a proxy for access), geographical location (urban or rural), and the monthly population size of the district where appropriate.

### Assessment of Inequality

WHO defines health equity as the absence of unfair and avoidable or remediable differences in health among population groups defined socially, economically, demographically, or geographically. In this study, inequalities in the coverage of MNCH services were measured and monitored and served as an indirect means of evaluating health inequity. We assessed the inequality of MNCH indicators between urban slum and non-slum districts using the Gini index with bootstrapped standard errors and the generalized Lorenz curve.

All statistical analyses were conducted using Stata MP version 17 (StataCorp LP, College Station, TX, USA) and a *p*-value less than 0.05 was considered statistically significant.

## Results

### Descriptive Analysis

This study analyzed 1392 monthly time-series data from 614 health facilities in 29 districts in Greater Accra that submitted data on coverage indicators to HMIS between 2018 and 2021. More than half of health facilities were privately owned (54%, *n* = 332), whereas government facilities and quasi-government facilities, including those operated by the Christian Health Association of Ghana, accounted for 42% (*n* = 255) and 4% (*n* = 27), respectively. About 76% of the total number of health facilities was in districts defined as having slums. The maximum number of slum locations per district was six (range, 0–6).

### Trend Analysis of Vaccination Coverage in Urban Slum and Non-urban Slum Districts

**Fig. 2:**
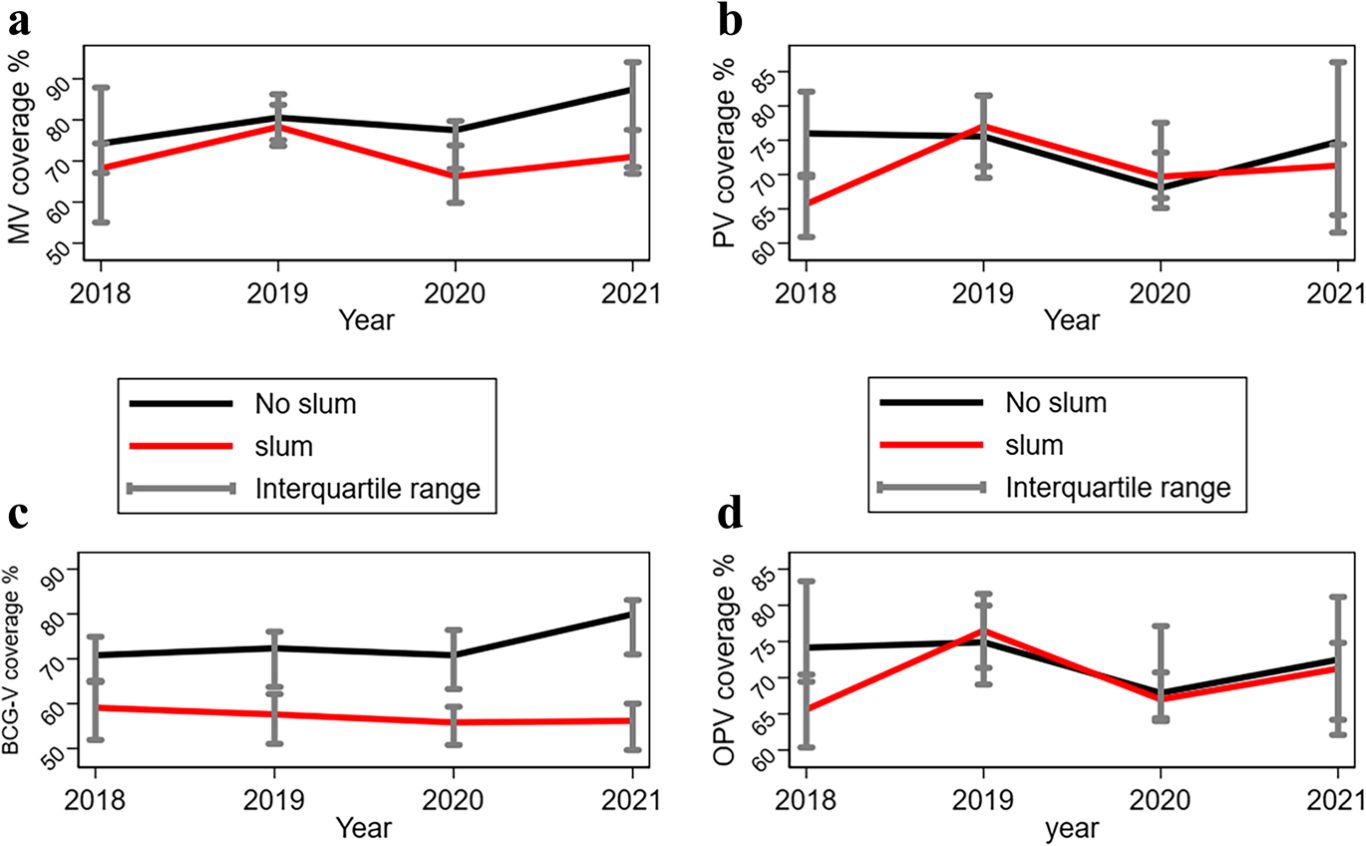
**a**–**d** Trend analysis of vaccination coverage in slum and non-slum districts from 2018 to 2021. Abbreviations: MV, measles vaccination; PV, Penta1 vaccination; BCG-V, Bacille Calmette-Guérin vaccination; OPV, oral polio vaccination

The trend analysis of vaccination coverage indicators showed that median BCG, Penta1, oral polio, and measles vaccination coverages declined in the urban slum districts compared to those in the non-urban slum districts in the Greater Accra region between 2018 and 2021 (Fig. 2). We observed a difference in oral polio vaccine coverage between urban slum and urban non-slum districts between 2018 and 2019, but the coverage gap decreased significantly between the two different types of districts from 2019 to 2021. Also, BCG coverage in urban non-slum districts was substantially higher than the coverage in urban slum areas.

### Trend Analysis of ANC, Skilled Delivery, and PNC Coverage in Urban Slum and Non-slum Districts

**Fig. 3:**
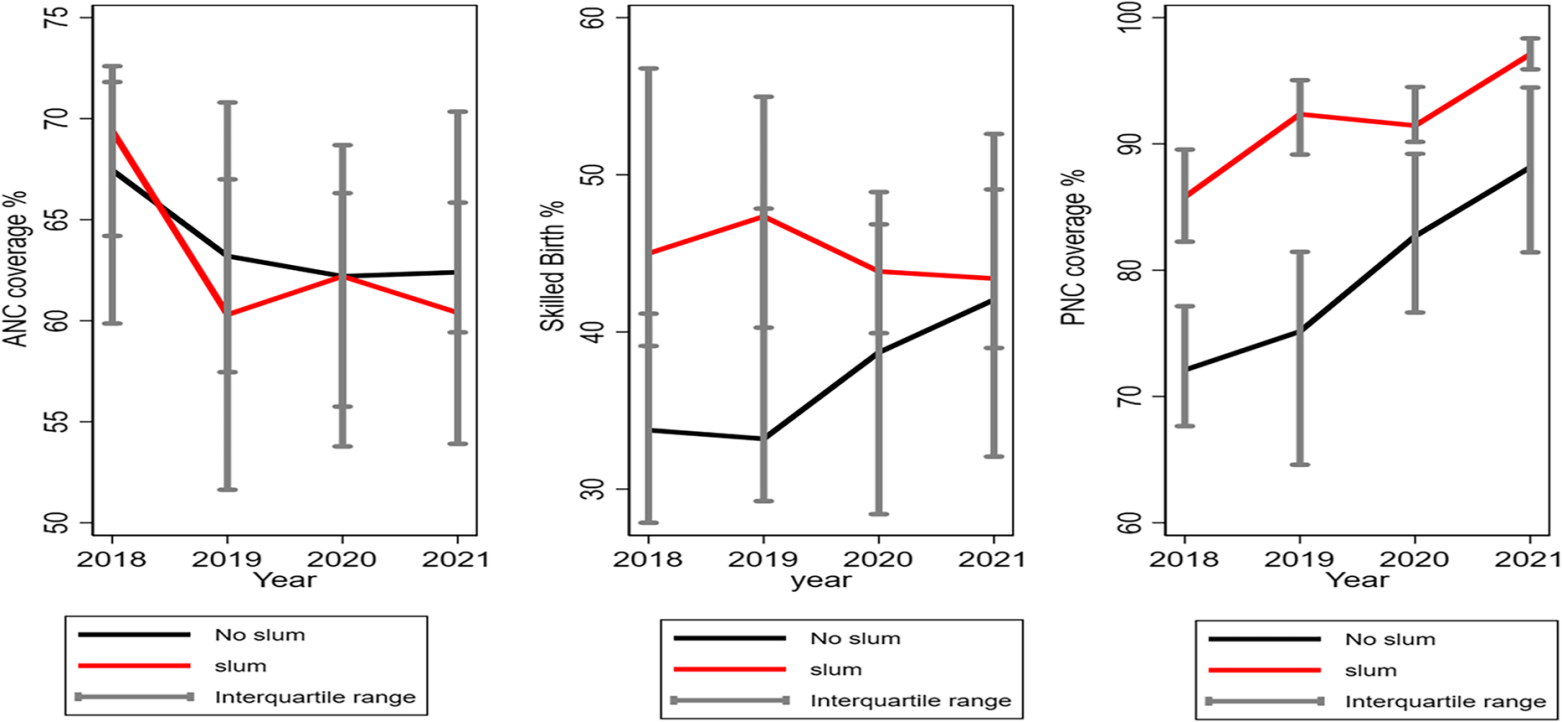
Trend analysis of ANC, skilled birth attendance, and PNC coverage in slum and non-slum districts from 2018 to 2021. Abbreviations: ANC, antenatal care; PNC, postnatal care

The trend analysis of coverage of ANC attendance, skilled birth attendance, and PNC attendance showed that median coverages of the indicators have declined in the non-slum districts compared to those in the urban slum districts from 2018 to 2021 (Fig. 3). ANC coverage appears to be declining in both slum and non-slum districts, but the decline appeared to be steeper in the slum districts compared to the non-slum districts over the same period. Although SBA appears to be higher in the slum districts, the gains appear to be eroding, while skilled attendance at birth is increasing at a higher rate in the non-slum districts. PNC coverage has consistently been higher in the non-slum districts compared to that in slum districts, and coverage appears to be increasing in both slum and non-slum districts.

### Impact of Urban Slum Residence on Health Service Coverage

The study found a statistically significant negative association between vaccination and health service coverage and living in urban slum districts in Greater Accra. The results from the quantile regression with robust standard error (Table 2) showed that a unit increase in the number of slums per district was associated with a 4.3 percentage point (pp) reduction in the BCG vaccination coverage [95% CI, − 5.87, − 2.79; *p* < 0.001], a 2.5 pp reduction in Penta1 vaccination [95% CI, − 3.86, − 1.14; *p* < 0.001], a 3.3 pp reduction in measles vaccination [95% CI, − 4.68, − 1.93; *p* < 0.01], a 5.4 pp reduction in oral polio vaccination [95% CI, − 6.87, − 3.99; *p* < 0.01], and a 2.6 pp reduction in ANC attendance [95% CI, − 4.35, − 0.89; *p* < 0.01]. Conversely, skilled birth attendance and PNC visits appear to have increased in slum districts compared to those in non-slum districts by 1.64 [0.50, 2.78] and 2.60 [2.06, 3.15], respectively.

**Table 2:** Impact of urban slum residence on MNCH service utilization in the Greater Accra region of Ghana

	Quantile regression modeling: reporting median impact estimates and corresponding confidence interval
Service coverage measures	*β* [95 % CI]
BCG vaccination	− 4.33 [− 5.87, − 2.79]***
Penta1vaccination	− 2.50 [− 3.86, − 1.14]***
Measles vaccination	− 3.31 [− 4.68, − 1.93]***
Oral polio vaccination	− 5.43 [− 6.87, − 3.99]***
ANC attendance	− 2.62 [− 4.35, − 0.89]**
Skilled birth attendance	1.64 [0.50, 2.78]**
PNC attendance	2.60 [2.06, 3.15]***

Abbreviations: *BCG*, Bacille Calmette-Guérin; *OPV*, oral polio vaccination; *ANC*, antenatal care; *PNC*, postnatal care; *CI*, confidence interval. *p*-value notations = ***p < 0.001, **p < 0.01, *p < 0.05

All the multivariable models adjusted for seasonality in month and year fixed effect, the impact of COVID-19, total outpatient department (OPD) attendance, number of health facilities in the district (a proxy for access), geographical location, and the population size of the district where appropriate

### Inequity Analysis of Coverage Indicators in Slum and Non-slum Districts

**Fig. 4:**
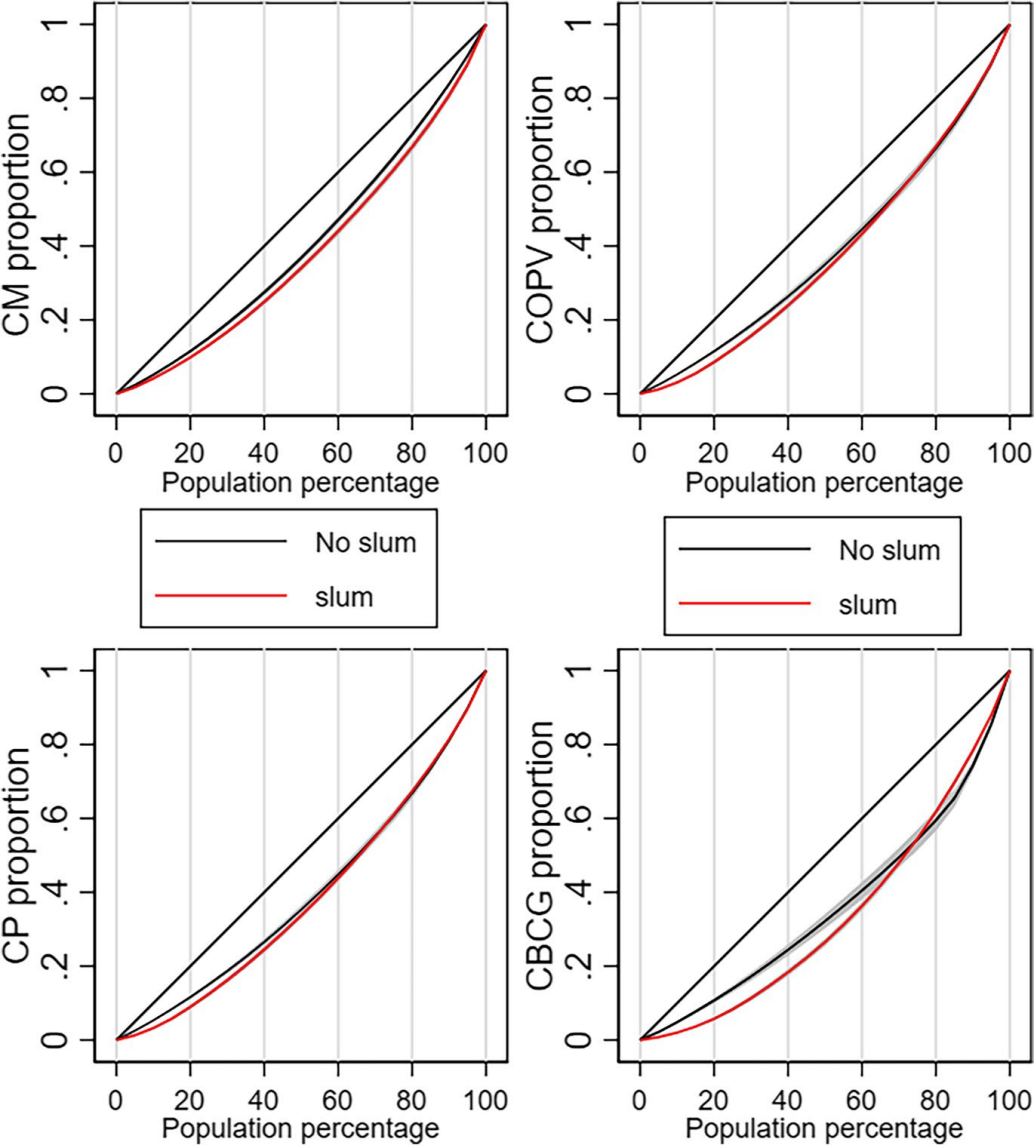
Health inequity assessment using generalized Lorenz curve. Abbreviations: CM, cumulative measles; CP, cumulative Penta1; COPV, cumulative oral polio vaccination; CBCG, cumulative Bacillus Calmette-Guérin

The Gini index, which ranges from 0 to 1, serves as a measure of inequality. In terms of service utilization, an index score of 0 signifies perfect equality in use, where service utilization is equally distributed among all groups. Conversely, a score of 1 indicates maximum inequality, where one group enjoys complete access to health services utilization while others have none. Inequality in BCG vaccination, measles vaccination, oral polio vaccination, pentavalent vaccination, ANC, and SBA was higher in slum districts compared to that in the non-slum districts. However, inequality in PNC was lower in slum districts compared to that in the non-slum districts (Table 3; Fig. 4).

**Table 3:** Inequality analysis of coverage indicators in slum and non-slum districts

	Slum status	
	Non-slum districts	Slum districts
Indicators	Gini index [95% CI]	Gini index [95% CI]
BCG vaccination	0.117 [0.107–0.127]	0.262 [0.246–0.278]
Measles vaccination	0.110 [0.099–0.122]	0.157 [0.147–0.167]
Oral polio vaccination	0.118 [0.110–0.127]	0.193 [0.180–0.207]
Pentavalent vaccination	0.117 [0.109–0.126]	0.185 [0.173–0.197]
Antenatal care	0.135 [0.124–0.146]	0.263 [0.247–0.280]
Skilled birth attendance	0.260 [0.242–0.278]	0.334 [0.315–0.352]
Postnatal care	0.143 [0.131–0.155]	0.115 [0.105–0.125]

The Gini index is a measure between zero (perfect equality) and one (maximum inequality), which in our case summarizes the degree of inequality in health service utilization between slum and non-slum districts in the Greater Accra region of Ghana between 2018 and 2021. A Gini index of zero indicates that everyone in the district has the same access, so there is perfect equality across the population. A Gini index of one lies on the other extreme and indicates that only one individual from the whole population has all the access, while everyone else has no access. Numbers closer to zero indicate less inequality, and the closer the Gini index is to one, the more unequal coverage is within the population considered

## Discussion

This study quantified the impact of living in slum districts on MNCH service coverage and explored the trend of usage of selected services in slum and non-slum districts, therefore allowing for comparison, over 4 years (2018–2021). Our findings showed that living in urban slum areas correlates with lower vaccination coverage and ANC attendance. However, the study revealed that people living in urban slums are more likely to report higher coverage of skilled birth attendance and PNC, which may be attributed to intense outreach programs carried out by government community health nurses and nongovernmental organizations (NGOs) focusing on the benefits of skilled birth attendance at health facilities and follow-up in the postnatal period. In addition, hospitals with high caseloads may be in slum areas where women from outside slums travel to seek the aforementioned services. For instance, the Ridge and Korle-Bu teaching hospitals are among the two most advanced health facilities in Ghana, and they are surrounded by slum communities. Since these are high-end facilities, they receive referrals, including the most critical cases, from health facilities located in other districts. The lower vaccination coverage in slum districts may be attributed to sociocultural factors [19, 20] and other misconceptions associated with vaccine hesitancy and lower ANC attendance since most childhood vaccination campaigns are emphasized during ANC [21]. These findings are similar to previous studies, albeit two different scenarios: Similar studies in Nepal and India that assessed the utilization of maternal health care services among women residing in slum areas concluded that utilization of ANC services was satisfactory but poor for postnatal and family planning services [3, 4, 22].

The equity assessment showed that inequalities in all coverage indicators except for PNC and skilled birth attendance were higher in slum districts compared to those in non-slum districts. The lower inequalities in PNC and skilled birth attendance coverage in slum areas may be attributed to a higher number of government health facilities (maternity homes, polyclinics, hospitals such as the Ridge and Korle-Bu teaching hospitals) in those areas. These facilities are all accredited by the National Health Insurance Scheme (NHIS), and access may largely not be a problem. In addition, under the new urban Community Health Planning Services (CHPS) initiative, community health officers are designated to urban zones, including slum locations, to provide MNCH services to urban poor communities.

Although there are a larger number of private health facilities in slum areas as well as non-slum areas in Accra, access to these facilities may be lower among urban slum dwellers compared to urban non-slum dwellers. This could be attributed to urban slum dwellers being poor and not able to afford services at private facilities in cases where those facilities do not accept NHIS due to reimbursement delays.

Vaccine uptake stagnated in both slum and non-slum district between 2018 and 2021. These findings are supported by other studies showing that vaccine acceptability has decreased in recent years, both in child and adult immunization programs, with serious consequences for public health [23]. Decreases in vaccination rates have contributed to increased prevalence of preventable childhood deaths and the reappearance of previously eradicated diseases such as polio and measles [24].

Concerns have been raised in print and electronic media, and on social media platforms, about the safety of vaccines, with many stories and claims based on myths, conspiracy theories, misinformation, disinformation, and rumors about the effectiveness and side effects of vaccines in preventive programs. Such misinformation and rumors promote fear and avoidance, thereby setting back progress made in addressing issues of vaccine hesitancy. In addition, urban slum areas by definition generally lack basic infrastructure and resilient health systems necessary to sustain vaccination campaigns geared toward demystifying myths associated with vaccination [25]. Slums are the geospatial manifestations of urban poverty, social neglect, and inappropriate and inadequate government policies. These factors are largely associated with lower service utilization and poor health outcomes [26]. There is a need for further studies to be conducted to determine factors influencing low vaccination coverage and ANC attendance in urban slum areas.

### Limitations

First, although the impact of COVID-19 was accounted for in the regression models, we believe the disruption to health service utilization was not fully captured, and this could potentially affect coverage patterns between 2020 and 2021 [27, 28]. Second, the analysis of data should have been conducted at the sub-district level, since the slum-impact variance could be higher at the sub-district level compared to the district level. This is because the size and intensity of the slum effect on households may differ across districts. For instance, a district may have only one slum, but the size of that single slum may be equivalent to a district with two slums located in different areas of the district. However, there were no reliable data sources with sub-district population size estimates that could be used to generate reliable denominators for the unbiased estimation of service coverage measures at the sub-district level. Third, the binary designation of districts as containing slums could suffer ecological fallacy as the proportion of slums in each district differs. Fourth, the differences in coverage of health service utilization between slum and non-slum districts could be affected by how we defined and categorized the districts into slum and non-slum, and this may have also impacted the results obtained from the inequality analyses. Fifth, the secondary data used to define slum areas might be out-of-date considering the rapid urbanization in Greater Accra. Sixth, the analysis does not control for “bypassing,” which refers to women living in non-slum districts accessing health services in slum districts and vice versa [29]. Finally, HMIS data tend to fluctuate and contain reporting errors. Therefore, fluctuations in service utilization should be interpreted with caution. Also, at the time of conceptualization and analysis, results from Ghana’s 2021 population and housing census were not yet available, so projections based on the 2010 census were used.

## Conclusion

Compared with non-slum districts, slum districts in Greater Accra have lower childhood vaccination coverage and ANC utilization but higher facility-based deliveries and PNC coverage. Accordingly, we advocate for a shift in the intensity of government policy implementation and interventions aimed at improving health service utilization among urban slum dwellers with a special focus on PNC and ANC. Interventions geared toward increasing vaccine uptake also should be a major government priority to address the stagnation in uptake of important childhood immunizations across districts.

## Data Availability

Data for the study are available upon reasonable request from Ghana Health Service (email: info@ghs.gov.gh).
